# Anemia in a Super-elderly Patient With Pelvic Fracture Complicated by Bacterial Translocation and Suspected Anti-neutrophil Cytoplasmic Antibody (ANCA)-Associated Vasculitis: A Case Report

**DOI:** 10.7759/cureus.92100

**Published:** 2025-09-11

**Authors:** Yudai Ozaki, Shiho Amano, Kurumi Kasai, Natsumi Yamamoto, Ryuichi Ohta

**Affiliations:** 1 Community Care, Unnan City Hospital, Unnan, JPN

**Keywords:** 80 and over, aged, anca-associated vasculitis, anemia, bacterial translocation, cutaneous, inflammation, leukocytoclastic, pelvic fractures, vasculitis

## Abstract

Anti-neutrophil cytoplasmic antibody (ANCA)-associated vasculitis is a systemic inflammatory disorder that may present with diverse clinical manifestations, including anemia of inflammation. We report the case of a 102-year-old woman who developed rapidly progressive anemia following a pubic fracture complicated by vascular injury. She presented with impaired mobility after a fall and was admitted for orthopedic management. During hospitalization, progressive anemia and fever were observed, prompting further evaluation. Imaging demonstrated a hematoma adjacent to the fracture site and ascites, raising suspicion of intra-abdominal infection. Laboratory studies excluded iron, vitamin B12, and folate deficiency, but ferritin was markedly elevated, suggesting anemia of inflammation. Empirical antibiotics were initiated for suspected bacterial translocation, resulting in clinical improvement. Additional evaluation revealed cytoplasmic-positive antinuclear antibody, elevated myeloperoxidase ANCA, and proteinuria, raising the possibility of ANCA-associated vasculitis as an inflammatory contributor. Due to her advanced age, invasive diagnostic procedures were not performed. Supportive management with transfusion and oral iron supplementation was combined with corticosteroid therapy, which led to improvement in hemoglobin and reduction in ferritin levels. She was subsequently transferred to a rehabilitation ward with the goal of eventual home discharge. This case highlights the diagnostic challenges of anemia in the super-elderly and underscores the need for pragmatic management when invasive investigations are not feasible.

## Introduction

Multiple traumas and severe complications often accompany pelvic fractures [1]. In particular, open pelvic fractures or complex pelvic ring injuries are associated with a high risk of infection and sepsis, which can significantly affect patient survival [2]. Recent reports indicate that the incidence of infections following pelvic fractures ranges from 7.5% to 14%, with higher rates observed in open fractures and cases requiring reoperation [3,4]. Once sepsis develops in the context of pelvic fractures, early identification of the infectious source and appropriate source control are essential to improve prognosis [5].

One of the mechanisms contributing to infection and sepsis in critically ill or injured patients is bacterial translocation. This process arises when intestinal barrier dysfunction allows gut bacteria or their products to cross the intestinal wall, spreading into mesenteric lymph nodes, the portal system, and systemic circulation [6,7]. Factors such as impaired tight junctions, ischemia-reperfusion injury, dysbiosis, systemic inflammation, and immune suppression all exacerbate gut permeability [8]. In critically ill patients, including those with major trauma or pelvic fractures, bacterial translocation may serve as an essential pathway leading to systemic infection, sepsis, and multiple organ dysfunction [8]. Although biomarkers such as circulating endotoxin have been investigated, diagnosis remains challenging, and prevention focuses on maintaining gut integrity through early enteral nutrition and careful systemic management [6,8].

Here, we report the case of a 102-year-old woman who sustained a pubic fracture complicated by right corona mortis artery injury and subsequently developed sepsis, likely facilitated by bacterial translocation in the setting of underlying gastrointestinal malignancy and chronic inflammatory disease. This rare clinical course underscores the importance of recognizing infection and sepsis risk after pelvic fractures, while also highlighting the complex decision-making involved in treatment strategies and discharge planning in super-elderly patients.

## Case presentation

A 102-year-old woman presented to a community hospital with impaired mobility after a fall. Imaging confirmed a pubic fracture, and she was admitted to the orthopedic service. She lived with her son and required long-term care (care level 3), supported by home nursing, helper visits, and short-stay services. During hospitalization, progressive anemia was observed, and the internal medicine team was consulted to investigate causes beyond trauma-related bleeding.

**Figure 1:**
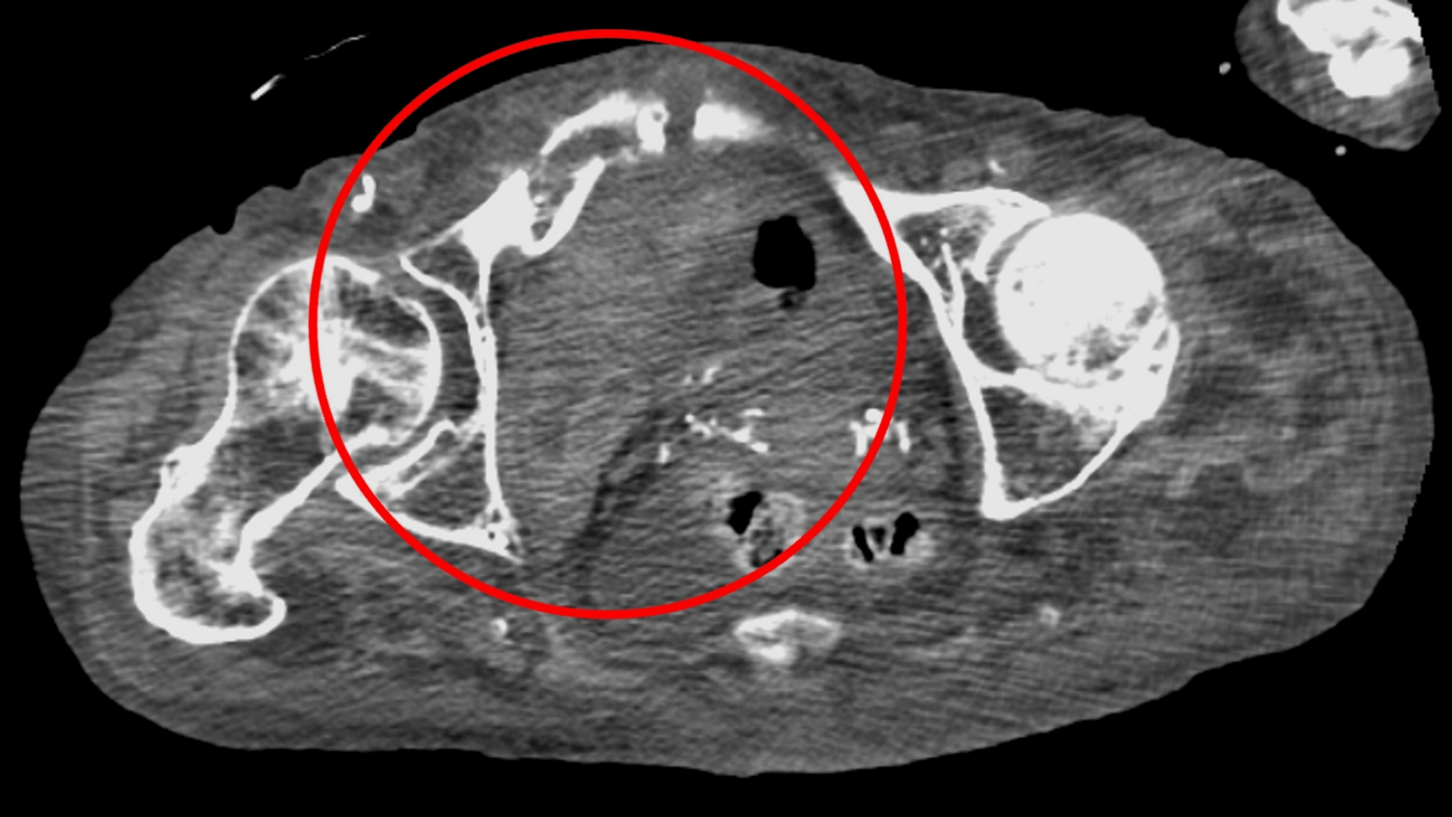
Contrast-enhanced computed tomography showing that the fracture was complicated by injury to the right coronary artery, with a hematoma near the fracture site that gradually enlarged (red circle)

The fracture was complicated by injury to the right corona mortis artery, with a hematoma near the fracture site that gradually enlarged on serial contrast-enhanced computed tomography (CT) scans (Figure 1).

Abdominal CT also revealed ascites. Around the same time, she developed fever, elevated inflammatory markers, and anorexia, raising suspicion of intra-abdominal infection and sepsis.

Her past medical history included Alzheimer’s disease, osteoporosis, chronic dizziness, gastroesophageal reflux disease, anemia, rheumatoid arthritis, and prior inguinal hernia repair. Medications included quetiapine 50 mg daily, esomeprazole 20 mg daily, eldecalcitol 0.5 µg daily, bisoprolol 2.5 mg daily, ferrous citrate 100 mg daily, and sennoside 24 mg as needed.

On admission, her blood pressure was 134/78 mmHg, pulse 76 beats per minute, respiratory rate 16 breaths per minute, body temperature 36.5°C, and oxygen saturation 96% on room air. She had pale conjunctiva, diffuse abdominal tenderness, and bilateral leg edema, more prominent on the right. Laboratory tests excluded iron, vitamin B12, and folate deficiency. Ferritin was markedly elevated at 1157 ng/mL, suggesting anemia of chronic inflammation (Table 1).

**Table 1:** Initial laboratory data of the patient Cl, chloride; CRP, C-reactive protein; K, potassium; Na, sodium

Parameter	Level	Reference
White blood cells	5.0 × 10^3^	3.5-9.1 × 10^3^/μL
Neutrophils	83.9	44.0-72.0%
Lymphocytes	9.0	18.0-59.0%
Hemoglobin	6.3	11.3-15.2 g/dL
Hematocrit	18.8	33.4-44.9%
Mean corpuscular volume	96.0	79.0-100.0 fl
Platelets	12.1 × 10^4^	13.0-36.9 × 10^4^/μL
Total protein	5.2	6.5-8.3 g/dL
Albumin	2.1	3.8-5.3 g/dL
Total bilirubin	1.7	0.2-1.2 mg/dL
Aspartate aminotransferase	14	8-38 IU/L
Alanine aminotransferase	4	4-43 IU/L
Lactate dehydrogenase	172	121-245 U/L
Blood urea nitrogen	30.2	8-20 mg/dL
Creatinine	0.72	0.40-1.10 mg/dL
Serum Na	144	135-150 mEq/L
Serum K	3.0	3.5-5.3 mEq/L
Serum Cl	105	98-110 mEq/L
Ferritin	1156.9	14.4-303.7 ng/mL
CRP	13.97	<0.30 mg/dL
Serum iron	124	50-170 µg/dL
Folate	14	2-20 ng/mL
Vitamin B12	268	200-900 pg/mL
Urine test	-	-
Leukocyte	Negative	Negative
Protein	2+	Negative
Blood	1+	Negative
Rod	Negative	Negative

**Figure 2:**
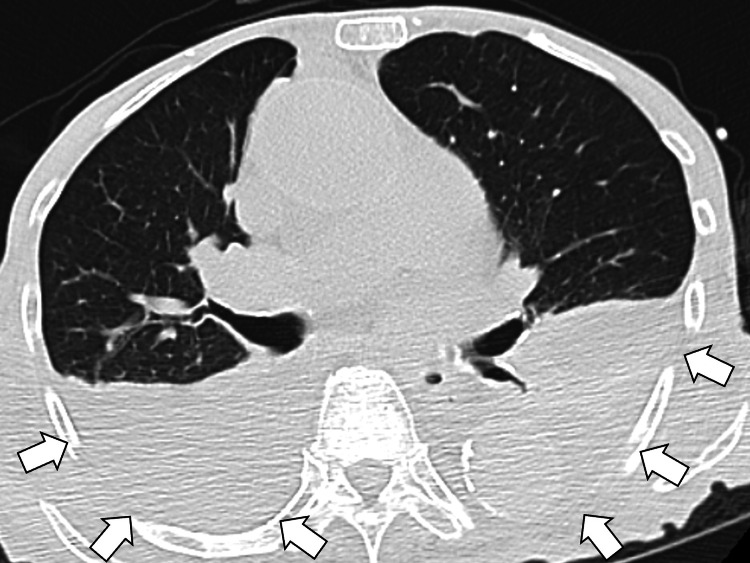
Computed tomography showing no gastrointestinal malignancy nor lymphadenopathy with bilateral pleural effusion (white arrows)

Fecal occult blood was positive, though no gastrointestinal malignancy was identified on CT with bilateral pleural effusion (Figure 2).

She received two units of packed red blood cells, and oral iron supplementation was continued, which improved her hemoglobin to 8.0 g/dL.

Given the presence of ascites and abdominal tenderness, intra-abdominal infection due to bacterial translocation was suspected. Empirical cefmetazole 4 g daily was initiated, resulting in rapid defervescence and improved oral intake. Antibiotics were continued for seven days with a good response. Blood cultures remained negative, echocardiography showed no vegetations, and a loop-mediated isothermal amplification (LAMP) test for tuberculosis was also negative. Tumor markers, including carcinoembryonic antigen (CEA), squamous cell carcinoma (SCC) antigen, and cancer antigen 125 (CA125), were negative. Autoimmune testing revealed a cytoplasmic-positive antinuclear antibody and elevated myeloperoxidase anti-neutrophil cytoplasmic antibody (MPO-ANCA) at 12.3 international units per milliliter (IU/mL) (reference <3.5), along with proteinuria. These findings suggested possible ANCA-associated vasculitis. Because the patient was extremely elderly, invasive procedures such as bone marrow biopsy or further invasive investigations were considered inappropriate and not performed.

Considering persistent inflammation, MPO-ANCA positivity, and proteinuria, ANCA-associated vasculitis was suspected as a contributor to her anemia. Prednisolone 10 mg daily was initiated. Two weeks later, hemoglobin improved from 8.0 g/dL to 11.2 g/dL, and ferritin decreased from 1157 ng/mL to 445 ng/mL. Oral intake improved, and her general condition stabilized. The overall course suggested that anemia was related not only to fracture-associated bleeding but also to systemic inflammation from ANCA-associated vasculitis. Corticosteroid therapy was effective, and her pelvic fracture was treated conservatively. She was transferred to a rehabilitation ward with the goal of eventual home discharge.

## Discussion

This case reports a 102-year-old woman who developed rapidly progressive anemia in the context of a pubic fracture complicated by vascular injury and subsequent sepsis, most likely due to bacterial translocation. The anemia was multifactorial, reflecting impaired iron utilization from chronic inflammation together with bleeding from coronary artery injury. Despite a comprehensive evaluation, the exact underlying inflammatory trigger remained uncertain. Because of her advanced age, invasive investigations such as bone marrow biopsy were not feasible, which limited diagnostic confirmation. Nevertheless, her clinical course highlights two important learning points.

Fever of unknown origin remains a frequent diagnostic challenge in elderly patients, who often present with atypical or nonspecific features [9]. Bacterial translocation - the passage of intestinal bacteria or bacterial products into the systemic circulation - has been increasingly recognized as a mechanism for sepsis and multi-organ dysfunction, particularly in critically ill or elderly patients with impaired gut barrier function [10,11]. In this patient, abdominal tenderness and ascites suggested an intra-abdominal source, and empirical antimicrobial therapy led to prompt improvement, supporting bacterial translocation as a likely contributor. Clinicians should not dismiss such cases as “unexplained fever” but instead consider bacterial translocation as a potential etiology. Early abdominal evaluation may uncover occult sources of infection or inflammation, enabling timely intervention. Recognizing bacterial translocation in the differential diagnosis may improve patient outcomes and reduce prolonged uncertainty for families and healthcare providers.

Anemia of inflammation (AI), also termed anemia of chronic disease, is characterized by impaired iron mobilization and restricted erythropoiesis mediated by inflammatory cytokines such as interleukin-6 and hepcidin [12]. The cornerstone of management is control of the underlying condition, while coexisting deficiencies of iron, vitamin B12, or folate should be corrected [13]. Erythropoiesis-stimulating agents may be indicated in selected patients, such as those with chronic kidney disease or severe anemia (hemoglobin <10 g/dL), but they require careful risk-benefit evaluation due to potential thromboembolic complications [14]. In the present case, iron deficiency was not identified, but blood loss from vascular injury was suspected to contribute to the anemia. Symptomatic treatment with oral iron and red blood cell transfusion stabilized her condition. The detection of elevated MPO-ANCA and proteinuria further suggested ANCA-associated vasculitis as an underlying inflammatory driver. ANCA-associated vasculitis can present with diverse systemic manifestations, including AI, and diagnosis in very elderly patients is challenging [15]. Given her age and frailty, invasive procedures were avoided, and corticosteroid therapy was initiated. Prednisolone at 10 mg daily successfully improved hemoglobin levels and reduced ferritin levels, supporting the control of inflammation as an effective therapeutic target.

General physicians in rural contexts can encounter older patients with anemia by ruling out infections, apparent malignancy, and hematological diseases. To improve their quality of life and facilitate their discharge to their home or long-term care facilities, the physicians have to manage their anemia without blood transfusion [16]. Autoimmunity pathophysiology is one of the reasons for anemia [17]. In this case, when general physicians detect the autoimmune features in older patients with mild to severe anemia, a trial of prednisolone may be practical in rural contexts [18,19].

## Conclusions

This case highlights the complexity of diagnosing and managing anemia in a super-elderly patient with multiple comorbidities. Rapid progression of anemia resulted from a combination of vascular injury-related bleeding, chronic inflammation, and possible bacterial translocation-induced sepsis. The additional detection of MPO-ANCA and proteinuria suggested ANCA-associated vasculitis as a contributing factor. Invasive diagnostic procedures were avoided due to advanced age, and treatment focused on supportive care and corticosteroid therapy. This pragmatic approach stabilized her clinical condition, emphasizing the importance of balancing diagnostic certainty with individualized, minimally invasive management in the very elderly.
